# Molecular Imaging of endometrial sentinel lymph nodes utilizing fluorescent-labeled Tilmanocept during robotic-assisted surgery in a porcine model

**DOI:** 10.1371/journal.pone.0197842

**Published:** 2018-07-02

**Authors:** Kristen M. Anderson, Christopher V. Barback, Zhengtao Qin, David J. Hall, Carl K. Hoh, David R. Vera, Michael T. McHale

**Affiliations:** 1 Department of Reproductive Medicine, Division of Gynecologic Oncology, University of California, San Diego, United States of America; 2 Department of Radiology, University of California, San Diego, United States of America; 3 UCSD Molecular Imaging Program, University of California, San Diego, United States of America; Wayne State University, UNITED STATES

## Abstract

Molecular imaging with a fluorescent version of Tilmanocept may permit an accurate and facile detection of sentinel nodes of endometrial cancer. Tilmanocept accumulates in sentinel lymph nodes (SLN) by binding to a cell surface receptor unique to macrophages and dendritic cells. Four female Yorkshire pigs underwent cervical stromal injection of *IRDye800*-Tilmanocept, a molecular imaging agent tagged with near-infrared fluorescent dye and radiolabeled with gallium-68 and technetium-99m. PET/CT scans 1.5 hours post-injection provided pre-operative SLN mapping. Robotic-assisted lymphadenectomy was performed two days after injection, using the *FireFly* imaging system to identify nodes demonstrating fluorescent signal. After removal of fluorescent nodes, pelvic and periaortic node dissections were performed. Nodes were assayed for technetium-99m activity, and SLNs were established using the “10%-rule”, requiring that the radioactivity of additional SLNs be greater than 10% of the “hottest” SLN. Thirty-four nodal samples were assayed *ex vivo* for radioactivity. All the SLNs satisfying the “10%-rule” were detected using the *FireFly* system. Five fluorescent nodes were detected, corresponding with preoperative PET/CT scan. Three pigs had one SLN and one pig had two SLNs, with 100% concordance between fluorescence and radioactivity. Fluorescent-labeled Tilmanocept permits real-time intraoperative detection of SLNs during robotic-assisted lymphadenectomy for endometrial cancer in a porcine model. When radiolabeled with gallium-68, Tilmanocept allows for preoperative localization of SLNs using PET/CT, and shows specificity to SLNs with persistent fluorescent signal, detectable using the *FireFly* system, for two days post-injection. In conclusion, these findings suggest that a phase I trial in human subjects is warranted, and that a long-term goal of an intra-operative administration of non-radioactive fluorescent-labeled Tilmanocept is possible.

## Introduction

An estimated 60,050 U.S. women will be diagnosed with endometrial cancer in 2016, with 10,470 deaths [1]. Uncertainty persists regarding the optimal surgical management of endometrial cancer, especially in clinically early-stage disease. Although the National Comprehensive Cancer Network (NCCN) Guidelines include hysterectomy, bilateral salpingo-oophorectomy (BSO), and nodal assessment, the rationale for and extent of lymphadenectomy remains controversial, specifically the limits of a para-aortic assessment [2].

The histologic assessment following a lymphadenectomy is utilized to define the need and type of adjuvant therapy (brachytherapy, whole pelvic radiation, chemotherapy or a combination of the two), but the therapeutic benefit remains controversial. Two randomized clinical trials were completed to assess the efficacy of lymphadenectomy in the management of endometrial cancer. Both trials failed to demonstrate a clear benefit in recurrence-free or overall survival following a lymphadenectomy, though many have criticized these trials for poor study designs and data analysis. As such, the controversy persists [3–5]. A more recent study including 476 patients with International Federation of Gynecology and Obstetrics (FIGO) stage IB to IIIC (intermediate or high-risk endometrioid-type endometrial cancer) noted a survival advantage in patients who had a more thorough pelvic lymphadenectomy. The number of negative pelvic nodes in this study was the most significant prognostic factor, even after controlling for age, grade, para-aortic node involvement, number of para-aortic nodes, total positive nodes, and adjuvant treatment [6]. The authors acknowledged that increased long-term morbidity is associated with a complete lymphadenectomy, including lymphedema and lymphocyst formation. Conclusions drawn from this study and others indicated that the survival impact of removing more nodes may stem from the staging accuracy and increased detection of metastasis. Variability in surgical technique and pathological evaluation of nodal tissue could compromise the generalizability of the results, however. Inclusion of a sentinel lymph node (SLN) mapping algorithm may help resolve this and other issues. Recent studies by Holloway, et al. [7] and Buda, et al [8] showed that SLN mapping with lymphadenectomy demonstrated increased sensitivity for detecting low-volume metastasis than lymphadenectomy alone, with a low false-negative rate. More recently, the FIRES trial, which assessed the use of indocyanine green for sentinel node mapping in patients with endometrial cancer, reported a sensitivity of 97% and successful mapping of at least one SLN in 86% of patients [9]. These results support the NCCN guidelines that in low-risk (type I) histology, SLN mapping, if successful bilaterally, may safely allow for the exclusion of a full lymphadenectomy [7, 8]. To date, no prospective randomized trials have established long-term survival data associated with SLN mapping in endometrial cancer.

Reducing the number of nodes removed by focusing on the specific nodes most likely to contain metastases will decrease the operative time, morbidity, and increased risks associated with full staging lymphadenectomy. Currently accepted SLN mapping techniques for endometrial cancer and cervical cancer involve cervical injection of blue dye, ^99m^Tc-labeled radiocolloid, Indocyanine green (ICG), or a combination of the tracers. Removal of any mapped nodal tissue in addition to any suspicious nodes is required, and a full lymphadenectomy must be performed on any non-mapped hemi-pelvis [10]. Ultra-staging is then performed on the identified sentinel nodes. An acceptable detection rate for sentinel nodes ranges from 80–90%, which differs significantly from the accepted rates (>98%) for melanoma and breast cancer. This difference is certainly linked to the need for bilateral mapping. The detection rates are also linked to surgeon experience [11, 12]. Improving mapping techniques, with more accurate identification of sentinel nodes, may minimize this variable.

Technetium [^99m^Tc]tilmanocept [13] (Tradename, Lymphoseek) is a radiopharmaceutical designed for rapid clearance from the injection site and sustained retention by SLNs. These attributes were are achieved by avid binding to a surface receptor on macrophages [14] and dendritic cells [15]. This molecular property provides specific and prolonged retention in the primary SLN without transport to secondary nodes [16]. Lymphoseek has been approved in the United States and Europe for sentinel lymph node mapping of breast cancer [17], melanoma [18], and head and neck cancer [19], with sensitivities of >97% for detecting SLNs. The head and neck SLN study used a pathology-based end-point, which yielded a false-negative rate of 2.6%.

Tilmanocept tagged with a near-infrared fluorescent dye [20] and the positron-emitting radionuclide, gallium-68, was developed as a tri-modal imaging agent [21] capable of providing quantitative preoperative PET/CT imaging, intra-operative fluorescent SLN mapping up to 72 hours after injection, and post-operative quantitation of SLN uptake. It has been evaluated in several animal models to date, and could identify SLNs of the hind paw of rats [14], rabbits [22], and prostate SLNs in dogs [23]. A more recent study performed by Lee, et al. [24], tested this method for identification of sentinel lymph nodes real-time during robotic-assisted cystectomy in a porcine model. The study examined 36 lymph nodes, detecting fluorescent-labeled tilmanocept using a standard fluorescence imaging endoscope up to 38 hours after submucosal injection into the porcine bladder. Utilizing the same method for endometrial cancer may improve the quality of lymphatic mapping and identification of the sentinel lymph nodes, limiting the need for extensive dissection and reducing surgeon-associated variation in the surgical management of this disease.

The primary objective of the study was to test the ability of the fluorescent camera, during robotic-assisted lymphadenectomy, to accurately detect sentinel lymph nodes after an endometrial injection of the molecular imaging agent. Ex vivo detection of radioactivity within each nodal bundle served as the “gold-standard” for SLN identification. Our short-term goal is a phase 1 clinical trial using preoperative imaging with PET/CT. Our long-term goal is the intra-operative administration of non-radioactive fluorescent-labeled Tilmanocept.

## Methods

### Study design

This study was designed as a non-survival study using 4 female Yorkshire pigs to evaluate a clinical protocol to identify sentinel lymph nodes in patients with uterine cancer several days after pre-operative injection of a multi-modal molecular imaging agent. The agent was tagged with a near-infrared fluorescent dye and radiolabeled with both gallium-68 and technetium-99m. Gallium-68 is a positron-emitting isotope with a 68-minute half-life, which permitted imaging via a positron-emission tomography (PET), and technetium-99m is a gamma-emitting isotope with a 6-hour half-life, which permitted a measurement of Tilmanocept SLN accumulation after completion of the robotic surgery. All activities in this study were reviewed and approved by the UCSD Institutional Animal Care and Use Committee.

### Cervical injection of tilmanocept and PET/CT imaging

**Fig 1:**
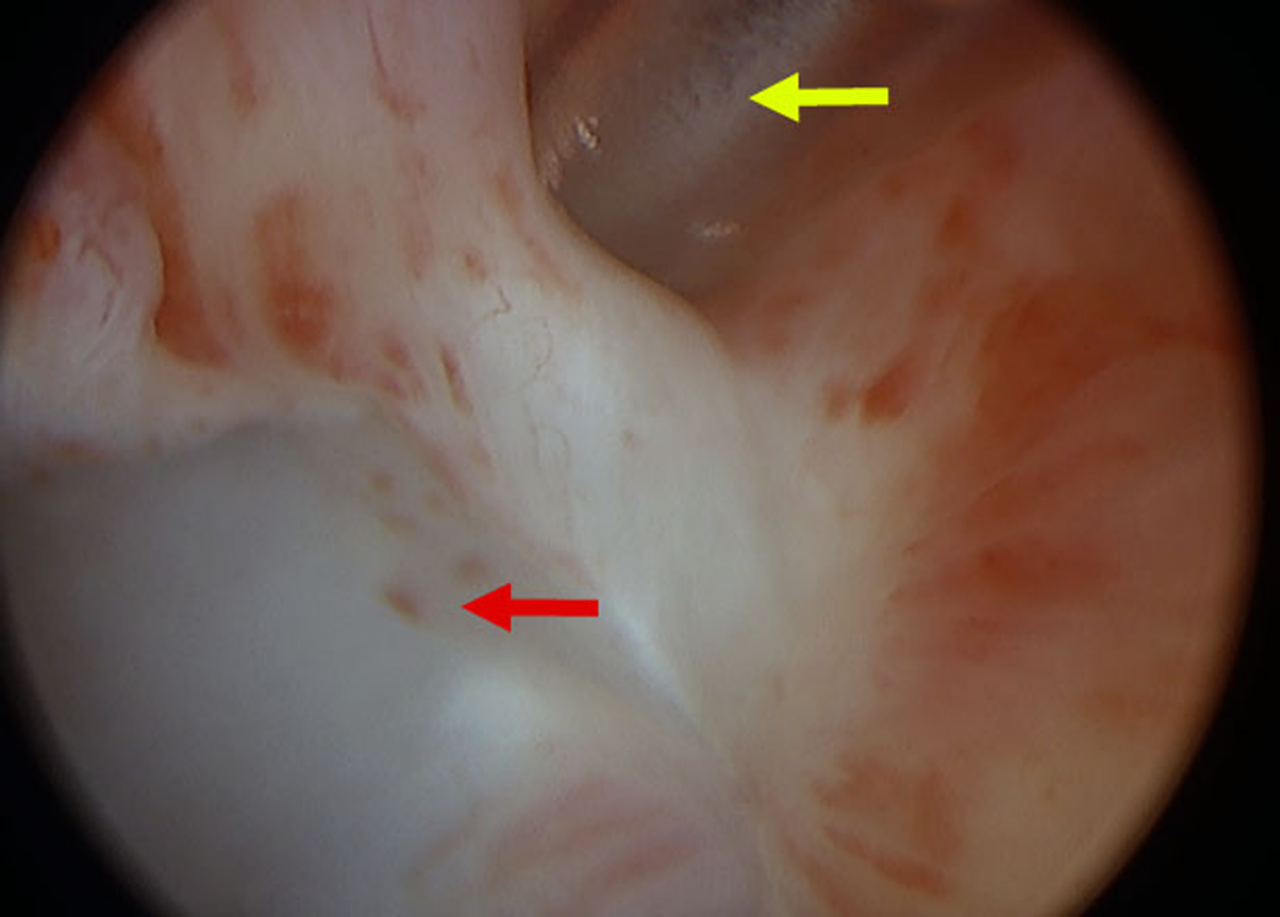
Cystoscopic administration of the molecular imaging agent. Injection of IRDye800CW-tilmanocept radiolabeled with gallium-68 and technetium-99m into the cervical mucosa (Yellow arrow indicates needle). Bluish tissue demarcates injection wheal.

The fluorescent dye *IRDye800CW* (LI-COR Biosciences, Lincoln NE) was covalently attached [20] to Tilmanocept (Navidea Biopharmaceuticals, Dublin OH) and radiolabeled [21] with Gallium-68 and Technetium-99m. Pigs were fasted overnight before each imaging and surgical procedure. During transportation to the imaging or surgical facility, each pig was sedated by intramuscular injection of an acepromazine/buprenorphine (0.10–0.05 and 0.01 mg/kg) cocktail. Under general anesthesia (propofol i.v., 2 mg induction, 10 mg/kg/hr maintenance), the pig was placed supine on the procedure table. A rigid cystoscope (Olympus America 19F, 12 degree lens, Center Valley PA) was inserted into the vagina. The vaginal fold, corresponding to the “cervical portio”, was identified. Using a botox injection needle (Coloplast Corp, Minneapolis MN), the molecular imaging agent, [^68^Ga][^99m^Tc]*IRDye800CW-*tilmanocept (1.5 nmol, 14.8–33.7 MBq ^68^Ga, ~7.5 MBq ^99m^Tc, 1.5 *IRDye800CW* dyes per tilmanocept, 0.75 mL total volume), was injected submucosally into four quadrants of the cervix at approximately 12, 3, 6, and 9 o’clock, forming a visible wheal at each injection site (Fig 1).

PET/CT imaging (Discovery VCT, G.E. Healthcare, Little Chalfont, United Kingdom) and acquisition protocols, previously described [23, 25], produced images of injection site, and the pelvic, and para-aortic lymph nodes. First, a scout CT was acquired to confirm the correct field-of-view. Next, a PET whole body scan (3 bed positions, 5 minutes per bed, 3D mode, and isotope selection set to “^68^Ga”) was obtained at 1.5 hours post-injection. Each PET image was followed by a CT acquisition (~1 second). Standard iterative reconstruction software was used to produce transaxial, coronal, and sagittal PET cross-sections, which were merged with the CT images to produce hybrid PET/CT images. SUVs for each visualized lymph node were calculated using GE *Volume Viewer* software (G.E. Healthcare). After injection and pre-operative imaging (day 0), the pig was awakened, transferred to an approved holding facility until day 2, when the pig was transported to the surgical research facility.

### Robotic-assisted surgery and *FireFly* fluorescent imaging

Approximately 40 hours after injection of the fluorescent-tagged radiopharmaceutical, a robotic-assisted surgical procedure was performed to identify and remove the SLNs and perform a full pelvic and para-aortic lymphadenectomy. After induction of general anesthesia, the pig was positioned in the supine position on the operating table. Using a Veress needle, the abdomen was entered and insufflated. A 15 mm laparoscopic port was then placed approximately 4 cm superior to the umbilicus, under direct visualization using a 10 mm laparoscopic camera. Three additional 8 mm and one additional 15 mm ports were placed in the standard manner for robotic-assisted pelvic lymphadenectomy. For the duration of the operation, the pig was placed in steep Trendelenberg position to allow the bowel to fall out of the area of dissection.

Using the PET/CT images as a guide, the *FireFly* fluorescence imaging endoscope (Intuitive Surgical, Sunnyvale CA) was used with the *da Vinci Si* robotic surgical system (Intuitive Surgical) to identify the SLNs. Peritoneal incisions were made near sites of PET avidity, and the fluorescence scope was used to carefully inspect the underlying retroperitoneal tissue for fluorescent signal. Once a fluorescent node was encountered, careful dissection was performed to isolate and excise the tissue. After the sentinel node was removed, the retroperitoneum was inspected and dissected in a systematic manner. The peritoneum was incised with monopolar scissors along the external iliac artery beginning just proximal to the bifurcation of the common iliac, and the incision extended cephalad parallel to the ovarian artery and caudally to the distal end of the artery. The para-vesical, para-rectal, and obturator spaces were developed. Using the *FireFly* fluorescence camera, each lymphatic basin was inspected for fluorescent signal. Complete bilateral pelvic lymphadenectomy, removing the remaining nodal tissue, was performed along the external iliac artery medial to the psoas and lateral to the ureter, along the internal iliac artery and vein, and from the obturator space anterior to the obturator nerve. The peritoneum was then incised along the aorta, just proximal to the bifurcation. The right and left common iliac arteries were identified, and the nodal tissue lateral to the aorta and surrounding and beneath the common iliac arteries was resected. Tissue removed from each site was collected separately and labeled as right or left pelvic or right or left para-aortic. After completion of the lymphadenectomy, the lymphatic basins were inspected again with the fluorescence camera. Once the surgical procedure was completed, the pig was euthanized.

### Post-excisional radioactivity and dye quantitation

The following lymph node bundles were excised and analyzed for technetium-99m activity: para-aortic, left and right common iliac, left and right pelvic, left and right obturator. Excised nodal tissue was collected in plastic scintillation vials and assayed with a gamma well counter for radioactivity. (100–200 keV window, Gamma 9000; Beckman Instruments, Fullerton CA). Counting standards with a known fraction of injected tilmanocept were analyzed and counts per minute determined. Empty scintillation vials represented the background count rate. These values were then compared to the counts per minute detected for each SLN and used to determine the fraction of the injected tilmanocept dose in each lymph node. To calculate the number of fluorescent dye molecules in each node, the average number of fluorescent dyes attached to each tilmanocept molecule (1.5 dyes per tilmanocept) was multiplied by the fraction of injected tilmanocept within the SLN and molar dose (1.5 nmol) of tilmanocept. The percent of injected dose (%ID) was calculated by standard methods [16].

## Results

Table 1 presents the details of the injection parameters used in each experiment. Also presented is the time between cervical injection and surgical lymphadenectomy, the number of SLNs identified by fluoroscopic visualization, number of nodes excised, and number of SLNs identified with *Ex-vivo* gamma counting of all excised nodes. The ^68^Ga dose ranged from 14.8–33.7 MBq. The time between injection and excision of SLNs ranged from 41–43 hours. Intra-operative SLN mapping was performed using the pre-operative PET/CT for guidance, and the *FireFly* camera successfully identified fluorescent lymph nodes corresponding to those present on PET/CT images in 5 of 5 locations. A total of 33 lymph nodes were excised and assayed for Technetium-99m activity and the “10% rule” for qualification as a SLN.

**Table 1:** Imaging and SLN mapping parameters.

	Molecular Imaging Agent*	Sentinel Lymph Nodes			Lymph Nodes
Study Number	^68^Ga (MBq)	Time between administration & SLN mapping (hr)	Number visualized by fluorescence during robotic surgery (n)	Number qualified by *Ex Vivo* gamma counting** (n)	Number of Lymph nodes Excised (n)
One	14.8	43	1	1	8
Two	33.7	42	2	2	7
Three	30.0	41	1	1	7
Four	17.8	41	1	1	11

* 1.5 nmol tilmanocept, ~7.5 MBq ^99m^Tc

** Using the “10%-rule” based on ^99m^Tc radioactivity measured in counts per minute

Table 2 lists, for each study, the SUVs of lymph nodes identified on PET/CT, the fluorescent status of the nodes identified by PET/CT, as well as the percent-of-injected dose and dye content of each lymph node in picomoles. Since there were no SLNs identified using the fluoroscope intraoperatively that were not visible on PET/CT, the percent-of-injected dose was only calculated for the fluorescent SLNs. Other nodal tissue demonstrated background gamma-counts of approximately 20 counts per minute, which was the number used as “background” for calculations. In each study, pre-operative PET/CT identified potential sentinel lymph nodes. Pre-operative SUVs identified 5 lymph nodes. SUVs of lymph nodes ranged from 11.9–56.0. Study three also identified a second area with an SUV of 84 that correlated with the position of the ureter and was not considered a SLN or listed in Table 2. The fluorescent signal did not persist at the time of surgery in this location, indicative of excretion from the GU tract.

**Table 2:** Quantitative data from excised lymph nodes.

Nodal Basin	Study One				Study Two				Study Three				Study Four			
	PET/CT	*FireFly*signal	Gamma Counter		PET/CT	*FireFly* signal	Gamma Counter		PET/CT	*FireFly* signal	Gamma Counter		PET/CT	*FireFly* signal	Gamma Counter	
	SUV	Y/N	% injected	Dye content (pmol)	SUV	Y/N	% injected	Dye content (pmol)	SUV	Y/N	% injected	Dye content (pmol)	SUV	Y/N	% injected	Dye content (pmol)
L Pelvic									56	Y	0.10	2.3				
R Pelvic																
L Para Aortic	22	Y	0.11	2.4	22.1	Y	0.18	3.9					11	Y	0.017	0.39
R Para Aortic					11.9	Y	0.11	2.5								

Of the four animals, three had one SLN and one had two SLNs identified intra-operatively. There were no additional fluorescent lymph nodes identified surgically that did not meet SUV criteria on PET/CT. *Ex-vivo* evaluation of the five SLNs detected with the fluoroscope demonstrated 100% concordance, for a total of 5 sentinel nodes meeting the “10%-rule” for technetium-99m activity. Percent-of-injected dose for fluorescent SLNs ranged from 0.017 to 0.18%, and the dye content ranged from 0.39–3.9 pmol, indicating a highly sensitive detection capability.

**Fig 2:**
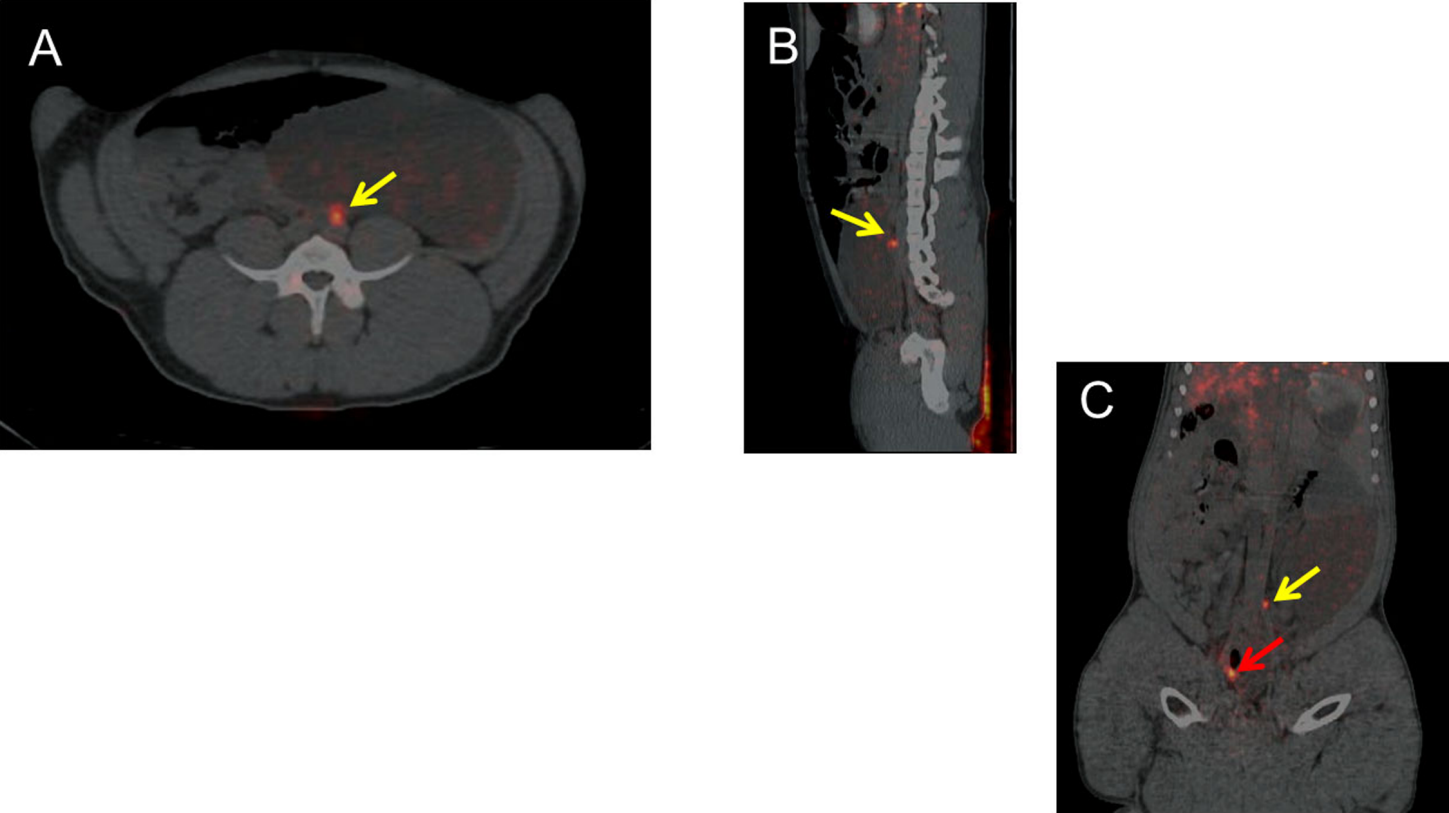
PET/CT imaging ninety minutes after administration of the molecular imaging agent. Fused PET/CT (A) transaxial, (A) sagittal, and (C) coronal views of the Left external iliac node, SUV 11. The injected dose of gallium-68 was 30.0 MBq. At the time of the PET/CT image, the SLN contained 3.0 MBq of gallium-68. There is an additional focus present on coronal view corresponding to the right ureter (red arrow).

**Fig 3:**
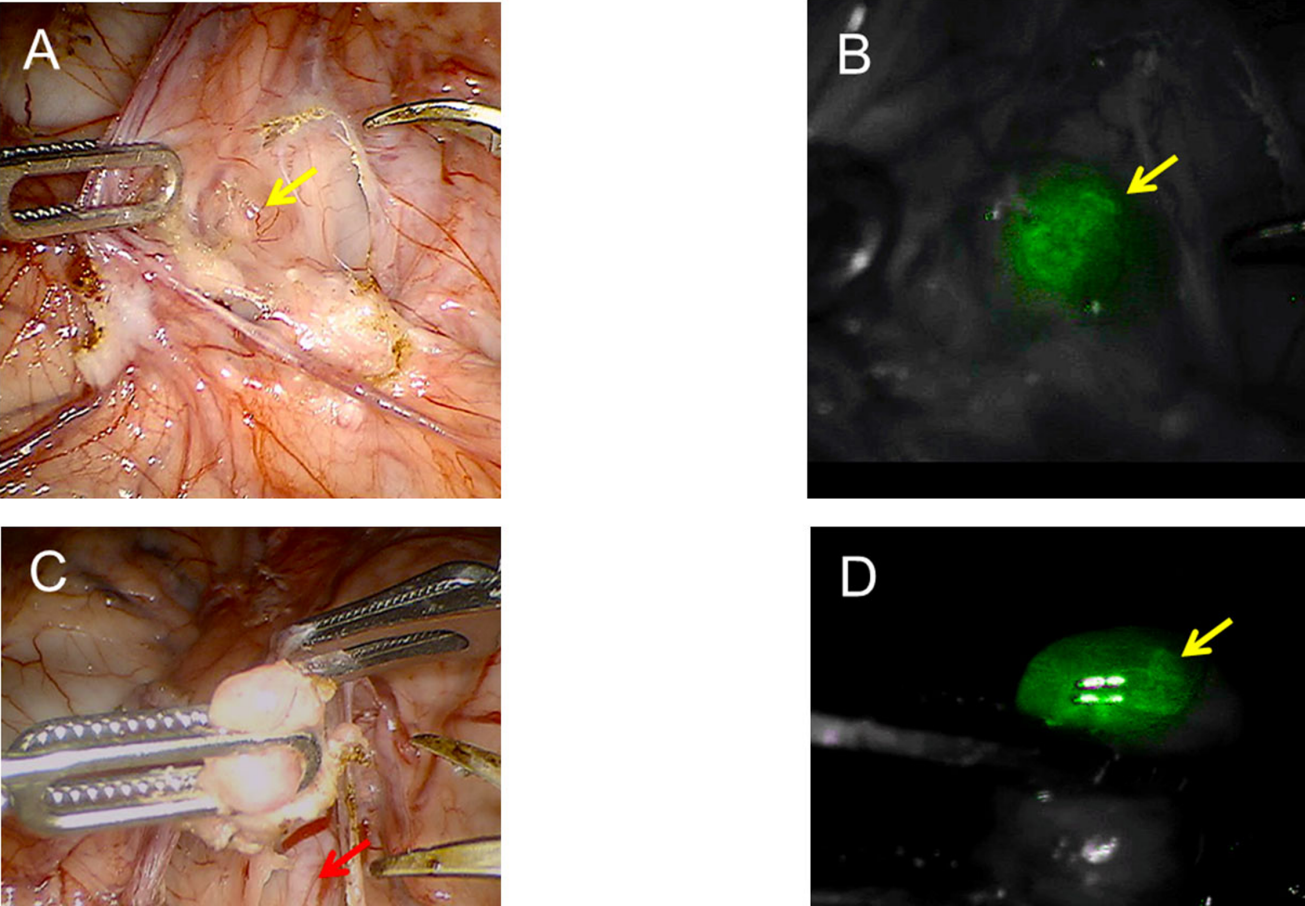
Intra-operative fluorescence SLN mapping 41 hours post administration. Intraoperative *FireFly* robotic surgical camera view with white light and corresponding fluorescent Near-infrared view before and after excision. This SLN contained 0.39 pmol of fluorescent dye. (A) White light, arrow indicates left pelvic lymph node prior to dissection. (B) Near-infrared in situ corresponding view of node. (C) White light post-dissection view of left pelvic node. Arrow indicates the left external iliac artery. (D) Near-infrared view of excised node.

In study three, Tilmanocept (1.5 nmol) labeled with 30.0 MBq ^68^Ga (Table 1) was injected superficially into four quadrants of the cervical stroma of a female pig using a cystoscope equipped with a botox injection needle (Fig 1), as described in the methods section. Approximately 1.5 hours after injection PET/CT scan was performed. Two areas of increased PET avidity were identified; one corresponding to the right ureter (SUV of 84), and another along the left external iliac artery (SUV of 56) (Fig 2). Forty-one hours after injection, robotic-assisted lymphadenectomy was performed., After positioning the surgical arms over the left external iliac lymph node basin, a fluorescent nodal packet was visualized when the *FireFly* camera system was switched to fluorescence mode (Fig 3 and S1 Video). After excision of this SLN, a full pelvic and para-aortic nodal dissection was completed, taking care to evaluate the nodal basins for fluorescence. No other areas of fluorescent signal were detected using the *FireFly* camera system. All of the nodal samples were placed in separate scintillation vials for radioactivity assay using a gamma counter. Seven nodes total were extracted (Table 2), but radioactive signal above baseline was only noted in the SLN. The percent of injected dose for this SLN was calculated to be 0.10%, and the fluorescent dye content 2.3 pmol (Table 2).

## Discussion

Results from this *in vivo* study demonstrated the capability of the *FireFly* fluorescence imaging system in identifying SLNs with fluorescent-labeled tilmanocept up to two days after intracervical injection. Use of this tri-modal radiopharmaceutical allowed for visualization of SLNs on pre-operative imaging, guiding surgical localization. Due to receptor-mediated sequestration, SLNs remained detectable in the sentinel lymph node using the *FireFly* fluorescence scope >40 hours after injection into the cervical mucosa, increasing the time frame for possible surgery. Most important, is that the number of SLNs detected by fluorescence matched the number of SLN identified by pre-operative imaging.

Currently the management of endometrial cancer, specifically the role for lymphadenectomy, continues to be controversial. The rationale and extent of lymphadenectomy has therapeutic or prognostic value, or simply may guide choice of adjuvant therapy [4]. Intraoperative decisions such as whether or not to perform pelvic and para-aortic lymphadenectomy and the degree of dissection remain surgeon-dependent and variable due to the interpretation of the existing data, surgical expertise, patient acceptance of risk, and patient risk factors [26].

Data supporting the use of SLN mapping techniques with an established management algorithm have prompted the NCCN guidelines [2] to include SLN mapping instead of full lymphadenectomy in select endometrial cancers with low risk of recurrence and in which full mapping is achieved. Lymphatic mapping to identify the sentinel lymph nodes, or the first nodes along the drainage route of a tumor, is becoming an acceptable practice in the management of low-grade endometrial cancer, though its application remains controversial. Optimization of this technique to improve sensitivity and specificity will further increase the utility of the practice with the goal of reducing morbidity imposed by a full nodal dissection.

Procedures included in the management of endometrial cancer remain controversial, but often include a full para-aortic and pelvic lymphadenectomy. Besides the increased morbidity, the degree of lymphadenectomy performed often differs according to surgeon and surgical skill. As a result, vastly different number of nodes are collected from case to case for analysis for metastasis. A larger number of nodes collected may increase the likelihood of removing the micrometastatic nests, but could reduce the detection rate if ultra-staging is not performed on all of the specimens. A validated technique with high sensitivity and specificity to identify SLNs, or the most high-risk nodes draining a tumor area, could reduce the need for extended lymphadenectomies and identify nodes that may be outside the usual dissection limits.

The molecular properties of tilmanocept distinguish fluorescent-labeled tilmanocept from ICG, blue dye, and radiolabeled particulates. Designed specifically for sentinel lymph node mapping, tilmanocept combines the best features of ICG and particulate-based imaging agents. Fluorescent-labeled tilmanocept localizes within lymph nodes based on a molecular interaction of extremely high affinity [14]. Consequently, Tilmanocept is retained by the sentinel lymph node for at least 24 hours in humans [16–19], and 36 hours in dogs [23] and rabbits [22]. ICG and blue dyes are incapable of binding to lymph nodes and consequently, are not retained by the sentinel lymph nodes. Radiolabeled particles are trapped by lymphatic tissue and can be used to detect SLNs may hours after administration. The entrapment is based on particle size and not a receptor-specific interaction. Tilmanocept is an order of magnitude smaller [13] than filtered Tc-99m-labeled sulfur colloid, the smallest radiolabeled particles available in the U.S. Consequently, Tilmanocept clears the administration site faster [27] than particles, accumulates at the SLN faster, and therefore, images SLNs immediately after administration [28]. Lastly, fluorescent Tilmanocept can be radiolabeled with a PET imaging radionuclide, Gallium-68. This bi-modal feature enables both PET/CT and fluorescence imaging. Although the final implementation of a Tilmanocept protocol for SLN mapping during robotic surgery may not employ radiolabeled tilmanocept and PET/CT imaging, PET/CT imaging will play a valuable role during the early phases of the clinical development (see Conclusion and future perspectives).

Being purposefully designed for sentinel lymph node mapping, tilmanocept will offer higher performance than the dyes and particles, which were introduced over 3 decades ago.

Papadia et al. [29] compared properties of ICG, blue dye, and Tc-99m-labeled particulates as they relate to sentinel lymph node mapping of endometrial cancer. The comparison was based on cost, ease of use, detection rates, patient comfort, need for additional equipment, and safety. The cost of bimodal tilmanocept will be higher than the dyes and radiolabeled particulates. However, the purchase of new instrumentation will not be required; PET/CT or SPECT/CT, as well as, surgical robots are common at regional medical centers. Fluorescent-labeled tilmanocept will not require specialize radio-detection equipment during the robotic or laparoscopic surgery. Although pre-operative tilmanocept imaging may not be used routinely, cross-sectional imaging may be extremely valuable in high BMI patients. Pre-operative images can direct the positioning of the robotic arms, shorten mapping time, and increase the confidence that a sentinel lymph node was not lost behind a fat layer.

Rapid and sustained sentinel lymph node accumulation will enable greater ease of use, high detection rates, and shorter learning curves for tilmanocept compared to the dyes and particles. The blue dyes and ICG rapidly travel through the sentinel lymph node and stain higher echelon non-sentinel lymph nodes. Retention of tilmanocept at the sentinel lymph node and low second echelon accumulation will enable next-day surgeries, if pre-operative imaging is needed. More important, the high SLN retention with provide greater integration of the mapping into the surgical procedure; this is especially important concerning the need to map bilaterally for endometrial cancer. If proven by a definitive clinical trial, the rapid and sustained sentinel lymph node retention of tilmanocept may eliminated the precautionary side-specific lymphadenectomy, which is currently part of the SLN mapping algorithm [30]. The hope is that a “designed for purpose” SLN agent will reduce the rate of side-specific lymphadenectomies from the 26% [31] to 58% [9] range to a value that one would expect for the incidence of true bilateral drainage. Lastly, the lack of second echelon staining, will reduce the number of lymph nodes sent to the pathologist.

Safety will also favor the molecular imaging agent. Fluorescent-labeled tilmanocept will be administered in microgram doses compared to the dyes, which require doses in the milligram range. As pointed out by Papadia [29], the blue dyes have a risk of severe allergic and anaphylactoid reactions; additionally, the 5% iodide content of ICG requires careful attention in patients with iodine hypersensitivity [11]. To date, over 200,000 doses of Lymphoseek have be administered to cancer patients without a single adverse event. Lastly, patient acceptance will favor tilmanocept; the injections are far less painful [32] compared to particles, and administered in volumes in the 0.1-ml range.

## Conclusion and future prospectives

The porcine model clearly demonstrated that fluorescent-labeled Tilmanocept permits real-time intraoperative detection of SLNs during robotic-assisted lymphadenectomy for endometrial cancer. The receptor-mediated retention of 48 hours after administration, warrants assessment by a Phase 1 clinical trial for SLN mapping in women with endometrial cancer. Future efforts will include a Phase 2 clinical trial to determine if a fluorescent-labeled tilmanocept can accurately map SLNs when administered immediately after anesthesia. Rapid SLN accumulation, which was exhibited by after intra-dermal [17] and oral [19] administrations, would eliminate the need for pre-operative administration and nuclear imaging. We envision the role of PET/CT imaging to be limited to the early phases of the development of our molecular imaging agent and high BMI patients. Wholebody PET/CT imaging during the Phase 1 clinical trial will document the molecular imaging agent’s biodistribution for regulatory registration. Pelvic PET/CT scanning during a Phase 2 trial will facilitate an understanding of the drainage patterns of endometrial cancer. The hopeful result will be a short learning curve and faster regulatory approval.

## Supporting information

S1 Checklist Arrive guidelines. Checklist for Animal Research: Reporting In Vivo Experiments.
(PDF)

S1 Video SurgeCut. In Vivo localization and dissection of a pelvic sentinel lymph node using Lymphoseek.
(MP4)

## Abbreviations

BSO: bilateral salpingo-oophorectomy
CT: computed tomography
FIGO: International Federation of Gynecology and Obstetrics
ICG: Indocyanine green
NCCN: National Comprehensive Cancer Network
PET: positron-emission tomography
SLN: sentinel lymph node
